# Application of state-target application of painful arthritis liver and kidney deficiency: A review

**DOI:** 10.1097/MD.0000000000031463

**Published:** 2022-12-02

**Authors:** Rui Zhou, Yuyan Jia, Ying Wang, Xukai Wang, Xiangyang Leng

**Affiliations:** a College of Traditional Chinese Medicine, Changchun University of Chinese Medicine, Changchun, Jilin Province, China; b The Affiliated Hospital of Changchun University of Chinese Medicine, Changchun, China.

**Keywords:** garbal arthritis, Si miao decoction, state-target syndrome differentiation, Tong Xiaolin

## Abstract

In recent years, with the progress and development of the times, our eating habits and lifestyle changes have led to an increase in gouty arthritis annually, with the main use of nonsteroidal anti-inflammatory drugs and other drugs. These drugs are highly dependent, resulting in an unresponsive state, which is easy to recur. Therefore, more and more patients choose traditional Chinese medicine (TCM) to treat them. After years of continuous exploration and rich clinical experience accumulation, Academician TongXiaolin put forward the dialectical strategy of “combination of state and target” in TCM. He believed that the deficiency of liver and kidney is transformed into a state, with uric acid as the target. Through the target prescription Simiao decoction to clever heat and moisture, replenishing liver and kidney, the target medicine Bixie (Dioscorea Tokoro Makino) to rheumatism, Shujin;Tufuling (Rhizoma Smilacis Glabrae) for detoxification, dehydration gas, Weilingxian (Radix et Rhizoma Clematidis) to rheumatism, pass meridians, and the combination of the condition and target achieves a good clinical effect.

## 1. Introduction

With the continuous improvement of the national economy, gout has become the most common joint disease in China and is related to morbidity and mortality. Crystalline arthritis caused by long-term deposition of uric acid monosodium salt in the body is directly related to hyperuricemia caused by decreased uric acid excretion or purine metabolic disorder, which belongs to the category of metabolic rheumatic diseases.^[1]^ The incidence of gout in China is increasing annually, and the incidence of gout in males is 8.6%.^[2]^ After investigation, it was found that patients often have a family genetic history, the onset age is above middle age, and most of them are men and postmenopausal women,^[3]^which has become a serious threat to the health of the people. Gout arthritis is often accompanied by clinical symptoms, such as sudden swelling and pain of toes, ankles and knees, stiff and deformed joints, relatively high skin temperature, local tenderness, limited movement, and so on.^[4]^ Western medicine mainly uses non-steroidal anti-inflammatory drugs, colchicine, glucocorticoids, and other drugs to treat this disease. These drugs have a good curative effect on gouty arthritis; however, they have serious side effects, high recurrence rates, and strong drug dependence.^[5]^ Therefore, the safe and effective treatment of this disease poses a challenge to traditional Chinese medicine (TCM). For a long time, through repeated clinical verification, the theory of “combination of state and target” by Tong Xiaolin has been proved to be targeted for the treatment of gouty arthritis, which not only has a significant effect, but also has the advantage of reducing adverse reactions.

There is no clear early literature record of gout in TCM, which falls into the category of arthromyodynia in the period of Internal Classic. The word gout was first seen in the famous Doctors’ catalogue: “It is only active, warm, and nontoxic. It is mainly used to treat various kinds of bandits, and there is no cure for gout in a hundred seasons.^[6]^” Zhu Danxi was the first to put forward the disease name gout in The Theory of Gezhiyu, the Theory of Gout. In Gezhiyulun Tongfeng, it is said that “people with gout have pain in hundreds of joints of 4 limbs, and the prescription book calls it Baihulijiefeng syndrome.^[7]^” It is believed that cold and heat intertwine, and cold blood leads to dirty coagulation block, pointing out that the causes of gout are mostly phlegm, heat, dampness, and blood deficiency.^[8]^ It is recorded in The Essentials of the Golden Cabinet—Syndrome and Treatment of Diseases and Pulse in Calendar Festival of Stroke that “the deep and weak pulse of sun you is the main bone, and the weak is the main muscle, and the deep is the main kidney, and the weak is the main liver...The yellow sweat in the calendar festival comes out, so it is called the calendar festival.” This indicates that the main reason for arthralgia syndrome caused by wind cold dampness is deficiency of the liver and kidney, which have lost some nourishment.^[9]^ Professor Chen Jifan believes that the fundamental cause of gout is deficiency of the liver, spleen, and kidney, while the weakness of tendons and cartilage due to deficiency of the liver and kidney is the place to retain pathogenic factors. If the spleen loses its function of health and transportation due to excessive fertilizer intake, or the spleen cannot absorb water and water due to spleen deficiency, damp heat will grow inside and flow into the joints, resulting in the loss of health care and nutrition and the loss of hardness in the stripes. Therefore, they are prone to the combination of exogenous damp heat, internal and external pathogens, and joint disorders, leading to gout.^[10]^ According to the opinions of TCM scholars, Academician Tong Xiaolin attributed gouty arthritis to improper diet, internal emotional injuries, congenital deficiency of liver and kidney, damp heat stagnation, blood stasis, dysfunction of spleen and kidney in ascending and clearing, and descending turbidity, etc. The above reasons will further block the circulation of qi and blood, and both of them are causal. After a long time, congestion and hair growth will cause gouty arthritis. Gouty arthritis has the characteristics of deficiency in essence and excess in reality, so it can be classified as “deficiency of liver and kidney, phlegm blocking damp-heat’.^[11]^

## 2. Methods

The theory of “combination of state and target” was first proposed by academician Tong Xiaolin. Academician Tong’s dialectical thought is based on the overall concept of TCM, and the main dialectical method is regulating state. The advantage of TCM lies in regulating the state. By taking advantage of the fact that drugs tend to cause diseases, they can balance the yin and yang, cold and heat in the body and fully exert the automatic regulation and repair ability of the body.^[12]^The “state” is constantly changing under a certain trend, which reflects the main aspects of the contradiction. We should take “illness” as the latitude and “state” as longitude. Improve the pertinence of treatment, so as to achieve a comprehensive grasp of the condition. Another layer of significance of “state-target combination” is the concept of “target,” which borrows from the concept of “target” in modern pharmacology.^[13]^ When we treat patients clinically, we take the abnormal pathological indexes of physical and chemical examinations and the most prominent symptoms of patients as “targets.” Microtargeting in modern pharmacology has greatly improved the accuracy of the clinical treatment of diseases, thus achieving ideal results.^[14]^ We have an absolute advantage in macro-control, but a serious deficiency in micro-targeting, which is directly related to the backwardness of natural life science research in TCM. Therefore, the theory of the combination of realm and goal proposed by Academician Tong is a brand new theory that combines the overall syndrome differentiation of TCM with modern medical indicators. The physical and chemical functions of TCM in modern pharmacology must be combined with the traditional dialectics of TCM, so that it is meaningful for modern pharmacology to return to clinical practice. The combination of state-target therapy highlights the efficacy advantages in the treatment of modern disease indicators, and is not only a clinical actual combat medical system, but also a guiding strategy for the innovation of TCM research.^[15]^

## 3. Discussion

The syndrome differentiation treatment of gouty arthritis is aimed at its “pathogenic state.” In addition to reducing acid as the root cause, reducing acid based on syndrome differentiation as the treatment state, and reducing acid based on symptoms as palliation, for the diagnosis and treatment of gouty arthritis, we should start with macroscopic adjustment of state, adjust the local state of the body, and keep the body in balance.^[16]^ Gout arthritis (liver and kidney deficiency type) is characterized by persistent pain, repeated attacks, soreness and weakness of the waist and knees, swelling and deformation of joints, mental fatigue, dizziness and tinnitus, red tongue with little coating, and a thready pulse.^[17]^ From the macroscopic analysis of deficiency of the liver and kidney in TCM, it can be understood that the kidney is the innate essence and the source of qi and blood generation, so it is called the natural origin. The kidney essence melts qi, and the kidney qi is divided into yin and yang. The kidney yang and kidney yin regulate the yin and yang of the zang-furgans in the whole body, so kidney is called the foundation of the 5 zang - fu organs. Su Wen’s “On Six Visceral Elephants” says that “the kidney governs pricking, which is the foundation of Tibetan.^[18]^”And pointed out that kidney is the foundation of Tibetan seal. The liver dredging, kidney is the innate foundation, and essence storage governs bone marrow. Under normal physiological conditions, the functions of liver dredging and kidney storing essence to control bone and bone marrow production are mutually restricted and opposite. If the functions of the liver governing dredging and draining and the kidney governing bone marrow production are out of balance, the physiological function of storing sperm will be affected.^[19]^ Liver and kidney deficiency syndrome refers to the coexistence of liver deficiency and kidney deficiency, with the liver and kidney from the same source, exuberant prosperity combined with exuberant weakness combined with weakness, as well as the inability of the kidney and water deficiency to nourish the liver, water failing to contain wood, and liver deficiency, or the transformation of qi into fire due to emotional turmoil. Or later stage of fever, consuming liver; liver injury easily involves kidney, forming liver and kidney deficiency. Based on this pathogenesis, Academician Tong believed that the “state” of gouty arthritis was the whole deficiency of liver and kidney. Therefore, the adjustment state is to fully considering that the patient is an organic whole formed at multiple levels, so as to achieve the desired result.^[20]^From a microscopic point of view, gouty arthritis is an inflammatory reaction caused by the abnormal purine metabolism in the body, which makes the excess uric acid deposited on the joints form crystals. Therefore, we focused on uric acid. The dialectical formula idea of “state-target combination” was used for analysis. Gout arthritis was treated by the regulation state” of the target prescription and drugs and by combining the state with the target.

Simiao decoction is a targeted prescription for tonifying the liver and kidney, strengthening bones and muscles, and eliminating dampness and turbidity. Simiao decoction is an important prescription for tonifying liver and kidney, strengthening tendons and bones, and it is also a target prescription for gouty arthritis due to liver and kidney deficiency. It is the first time for Zhang Bingcheng to record Simiao Powder in his book “Reading into Convenience.” Zhang Bingcheng said: “Although the evil of damp heat thrives in the lower jiao, it must originate from the spleen and stomach … the disease spreads in the lower jiao, but if it is treated in the middle jiao, it is incurable.^[21]^” This prescription can not only clear away heat, dry dampness, dispel stagnation, and relieve pain, but also has the effect of tonifying liver, spleen, and kidney. So as to achieve that purpose of eliminating pathogenic factor without hurting the right and strengthening the body resistance without removing pathogenic factors. Simiao decoction is made on the basis of Ermiao pill and Sanmiao pill. Ermiao Pill is composed of Rhizoma Atractylodis and Cortex phellodendri, with the main effects of clearing away heat and dampness and cleaning up the damp heat in the lower energizer. The Sanmiao pill is made by adding Achyranthes bidentata based on the Ermiao pillar, which can be used to induce fire, reduce adverse effects, nourish the liver and kidneys, strengthen muscles and bones, and treat lower jiao weakness.^[22]^ Coix lachryma-jobi is gentle in nature and can strengthen the spleen and stomach. Therefore, these 3 prescriptions can be described as coming down in the same strain: Cortex Phellodendri in Simiao decoction tastes bitter and cold; the kidney and bladder meridian entered; it has the effects of clearing heat and drying dampness, purging fire and detoxification, and removing bone steaming. It mainly enters the kidney meridian, and is good at removing the damp heat in the lower jiao, and clearing away heat due to bitter cold. The cold can destroy heat, and the bitter can dry heat and destroy heat. It is used to treat diseases such as damp heat flowing down the bladder and bone steaming and hot flashes, so it is a monarch drug. Atractylodes lancea has a pungent, bitter, and warm sexual taste; it flows into the stomach, spleen, and liver meridians; it dissipates cold, expels wind, eliminates dampness, and invigorates the spleen. Direct access to Zhongzhou. The spleen belongs to the soil of the 5 elements and has the physiological characteristics of liking dryness and hating dampness. It can be used for treating edema and dysuria caused by spleen and stomach qi deficiency and is especially effective in invigorating the spleen and stomach, eliminating dampness and diuresis, and is therefore a ministerial medicine. Coix lachryma-jobi is sweet, light, and cold in nature, and enters the meridians, lung, stomach, and spleen; it has the effects of diuresis, dampness elimination, spleen strengthening, and dampness elimination. It can enter into Yangming River alone, remove damp heatnd promote the movement of tendons and collaterals, govern the movement of tendons, and guide the movement of damp heat out of the stool as an adjuvant drug. Achyranthes bidentata tastes sour, sweet and bitter; liver and kidney meridians entered. It can tonify the liver and kidneys, strengthen tendons and bones, promote blood circulation, dredge channels, and induce fire to descend. It can be used to remove damp heat by descending the lower jiao, and can be used for both the liver and kidney.^[23]^ It is useful for treating joint pain. When used with the first 3 herbs, it tonifies the liver and kidneys, inducing diuresis and dredging stranguria. The combination of these herbs enables the release of damp heat and elimination of impediment syndrome.

According to the Pharmacopoeia of the People’s Republic of China (2020 edition), *Atractylodes lancea*, *Achyranthes bidentata*, *Cortex Phellodendri*, and Coicis Semen are the 4 medicines that make up this recipe. Has the effects of clearing away heat and promoting diuresis, and can be used for treating rheumatism caused by downward flow of damp heat, with the symptoms of red and swollen feet and knees and pain of bones and muscles.^[24]^ Modern pharmacological research showed that, Rong, etc.^[25]^ It is believed that PTGS 2, PPARG, CCL 2, IL-6, and CXCL 8 were the key genes for the treatment of gouty arthritis with Simiao decoction. The important active components of Simiao decoction were quercetin, kaempferol and wogonin, all of which were flavonoids. Quercetin plays an anti-inflammatory role by reducing the levels of inflammatory factors, while kaempferol reduces the formation of uric acid by competitive binding with xanthine oxidase, a key enzyme in purine catabolism.^[26]^ Zhang Yan^[27]^ found that Simiao decoction had 16 active components in the treatment of gouty arthritis. Among them, berberine, as the most effective component, inhibits the differentiation and function of osteoclasts by inhibiting the phosphorylation of NF-kB in osteoclasts precursors, osteoblasts and mature osteoclasts, and exerts analgesic, antipyretic and anti-inflammatory effects by interfering with mitogen-activated protein kinase, inhibiting the activity of nuclear factors and the secretion and release of inflammatory factors. A number of research results show that Simiao decoction is flexible in clinical application, especially for the targeted treatment of gouty arthritis.

Bixie, Tufuling, and Weilingxian can be used to assist in regulating the state of Simiao decoction, and can also be targeted. Bixie tastes slightly bitter and cold, and has the effects of clearing away heat, eliminating dampness, dispelling wind and dampness, relaxing muscles and tendons, activating collaterals, and dredging collaterals.^[28]^ In Compendium of Materia Medica, it is recorded that Rhizoma Dioscoreae Septemlobae is a kind of medicine for the foot-Yangming and Jueyin meridians. Jue Yin main reinforcement belongs to the wind, Yang Ming main meat is wet, the work of Bi XieZhi, good at to rheumatism, so can smelting treatment slow weak stubborn bi, heritage turbidity, evil sores of rheumatism.^[29]^ Tufuling is sweet, light, and even, and has the effects of removing toxins, detumescence, dampness, and dredging pulse.^[30]^ It is recorded in “Materia Medica Justice” that Tufuling can clear away heat, eliminate dampness, remove heat, and dredge collateral to eliminate cold and dampness.^[31]^ It is recorded in Materia Medica of Southern Yunnan that Tufuling is capable of “strengthening spleen and stomach,^[32]^ strengthening bones and muscles, eliminating rheumatism,^[33]^ and promoting joint movement,^[34]^” and it is combined with Bixie and Weilingxian to remove dampnes,^[35]^ dredge pulse, relax tendons, and activate collaterals.^[36]^It is recorded in the Book of Materia Medica that “all winds make good deeds, and those who do good deeds should also be guided by the herbs that are the root of wind...Wind energy wins wet, wet and dry, so it is the main system.^[37]^”In the Compendium of Materia Medica, Weilingxian is recorded as the main medicine for gout, which is suitable for both upper and lower levels.^[38]^ It is also named as a specific medicine for gout and intractable rheumatism in the application of medicinal cutting.^[39]^ Xu et al^[40]^ summarized the actual clinical application and dosage experience of radix clematidis by famous contemporary physicians and concluded that the dosage of the clinical decoction was generally 10 to 30 g, and the large dose could be up to 30 to 50 g. Academician Tong Xiaolin summed up the clinical experience and concluded that the dosage of Clematis chinensis was 15 to 30 g.^[41]^ No adverse reactions were noted. It is the core target drug of the academic Tong Xiaolin for the treatment of gouty arthritis.

In terms of targeting, studies have shown that Bixie contains a variety of steroidal saponins such as dioxin, and has anti-infection effects. It can improve hypermetabolism of uric acid and reduce the level of uric acid in blood.^[42]^ Pharmacological studies have confirmed that total saponins of Dioscorea zingiberensis have the effect of reducing uric acid levels. Wang et al^[43]^ studied the mechanism of total saponins of Dioscorea septemloba on gouty arthritis in rats and found that the expression levels of IL-1, ILIL-18d TNF-TNF-erum in rats were significantly increased, suggesting that IL-1β, IL-18, and TNF-α play an important role in the acute attack of gout, which was stronger than the alcohol extract of Dioscorea septemloba.^[44]^ Tufuling has anti-infection and immunity-regulating effects and can promote the metabolism of high uric acid and reduce the level of blood uric acid. Modern pharmacological studies have shown that resveratrol, an active ingredient of tufuling, can inhibit the activity of xanthine oxidase,^[45]^ enhance the antioxidant capacity of the body, and reduce purine catabolism, thereby inhibiting the generation of uric acid.^[46]^ Modern pharmacological research has revealed that the triterpenoid compounds in Weilingxian have antibacterial and anti-inflammatory effects. Peng et al^[47]^extracted the triterpenoid compound (AR-6) from radix clematidis and administered it to rats via gavage. The results showed that the 3 signaling pathways of TNF-α, PI3K, and p-Akt were all inhibited,^[48]^while the mRNA level in the synovium of rats decreased, which suggested that triterpenoids in Clematis chinensis could block the inflammatory reaction through the PI3K/Akt signaling pathway. Deng et al^[49]^ showed that total saponins of Clematis chinensis could inhibit the activation of the JAK2/STAT3 signal pathway, thus achieving an anti-inflammatory effect.

### 3.1. Case

XX, male, aged 44, was first diagnosed on January 3,2022. Chief complaint: In the past 4 years, there have been repeated toe-to-finger joint pain, which worsened for 3 days. Past history: 4 years ago, after eating barbecue and drinking a lot of seafood, our patient developed swelling and hot pain in the first metatarsophalangeal joint. After several days of continuous treatment, he spontaneously relieved the pain without paying attention and gradually affected all joints of the toe and finger with joint dysfunction. When pain occurs, the patient cannot bend or extend. He was diagnosed with gout at an outer hospital and often received comprehensive treatments, such as colchicine, glucocorticoid, febuxostat, indomethacin, and joint local hormone block. The patient failed to achieve any remarkable therapeutic effects. Serum uric acid was at a high level of approximately 500umol/L for a prolonged period. Three days prior, after drinking again, the preemptive symptoms became more obvious, and the patient presented to our hospital with a total white blood cell count of 12.20 × 109/ L, neutrophil count of 9.06 × 109/ L, and blood uric acid of 700.5 umol/L. Lettering disease: Unbearable swelling and pain of joints, mental fatigue, dizziness, tinnitus, soreness of the waist and knees, self-complaint, disturbing heart, loss of appetite, poor sleep, and constipation. Red tongue, little fur, and pulse string number.

Western Medicine diagnosis: Gouty arthritis. TCM diagnosis: arthralgia (liver and kidney deficiencies). Treatment principle: nourishing the liver and kidney and clearing dampness and turbidity. Medicine: Astragalus membranaceus 10 g, Clematis chinensis 10 g, Atractylodes lancea 10 g, Achyranthes bidentata 10 g, Cortex Phellodendri 10 g, Coicis Semen 10 g, Rhizoma Dioscoreae Septemlobae 15 g, Smilax China 10g and Semen Ziziphi Spinosae 30 g. 14 doses, 1 dose/d, patients are required to take it after supper and before going to bed.

The patient returned to our hospital on January 17, 2022, he returned to our hospital. In our patient’s self-reported case, the phalangeal joint of her toe improved without further pain and her blood uric acid level was 372 µmol/L. The patient was in a good mood, slept, and recovered physically. In order to achieve better effect, the patient took the medicine for 14 days continuously, and the blood uric acid basically returned to normal. He was also told to eat less food with high purine content, refuse to drink alcohol, keep a happy mood, and avoid overwork. After 1 month, the patient was followed-up by telephone. The patient’s condition was stable, and the overall quality of life improved significantly (Table 1).

**Table 1:** Comparison of test results before and after treatment.

Before treatment	Result	After treatment	Result
WBC	12.20 × 10^9^/L	WBC	6.30 × 10^9^/L
NEUT%	9.06 × 10^9^/L	NEUT%	4.10 × 10^9^/L
Cr	116.30 umol/L	Cr	91.30 umol/L
Urea	9.0 mmol/L	Urea	7.50 mmol/L
UA	700.5 umol/L	UA	372 umol/L

## 4. Conclusions

According to the patient’s long-term drinking and food with high purine content, his toes and fingers had joint pain, and his blood uric acid was high. He often took colchicine, glucocorticoids, febuxostat, and indomethacin, resulting in impaired liver and kidney function. The origin was the same as that of the liver and kidneys. The kidney is not enough to nourish the liver, and the water does not contain wood, which will damage the liver. Liver injury easily affects the kidneys; combined with the symptoms and tongue and pulse manifestations, it is known that the total pathogenesis of patients is a deficiency of the liver and kidney. Liver and kidney deficiency, fatigue, dizziness, tinnitus, soreness of the waist and knees, and swelling of the toe joints. Academician Tong Xiaolin believed that the core pathogenesis of tongue pulse, combined with the symptoms such as fatigue, dizziness and tinnitus, soreness of waist and knees, and joint pain, could be summarized as deficiency of liver and kidney. Therefore, we chose Jiawei Simiao Decoction. Therefore, the original prescription of Simiao Decoction is mainly based on regulating the state. As for target practice, the cortex phellodendri in the prescription has antibacterial and anti-inflammatory effects to inhibit the inflammatory mediators of joints during gout attacks. In addition, emodin can inhibit the activity of xanthine air oxidase, thus further affecting the production of uric acid and reducing its level of uric acid in the blood. In addition, Phellodendron amurense also protects the kidney and regulates kidney deficiency, and the 2 medicines Rhizoma Atractylodis and Cortex phellodendri not only have the compatibility of the TCM prescription pair for clearing heat and removing dampness in terms of TCM theory, but also have an outstanding effect of reducing uric acid in modern medicine. Coix seed also has targeted anti-inflammatory and analgesic effects and can promote the excretion of uric acid and reduce uric acid levels. Achyranthis Radix has the effect of protein assimilation, and the ecdysterone contained in it can promote the synthesis of proteins relatively strongly and has the function of dredging joints and detoxification. Bixie removes dampness and turbidity, and expels wind and bi. Weilingxian dispels wind and eliminates dampness. Modern materia medica has proven that Tufuling, Bixie, and Weilingxian have good effects in eliminating dampness, and they are typical “state-target combination” prescriptions.^[38]^ The patient was upset, had poor sleep, and felt tired; therefore, Astragalus membranaceus and Semen Ziziphi spinosae were selected in the prescription. Huangqi (Radix Astragali seu Hedysari) is a tonic that invigorates qi, elevates yang, induces diuresis, supports toxins, drains pus, and promotes tissue regeneration. Semen Ziziphi Spinosae nourishes yin, nourishes blood, tranquilizes the mind, and resolves the mind.

## Author contributions

XL and XW conceived and designed the work; XL and XW helped to coordinate support and funding; RZ, YJ, and YW conduct data statistics; RZ analyzed the data and wrote the original draft; XL and XW reviewed and revised the manuscript. All authors read and approved the final version of manuscript.

**Conceptualization:** Yuyan Jia, Xukai Wang.

Data curation: Rui Zhou.

Formal analysis: Yuyan Jia.

**Investigation:** Rui Zhou, Yuyan Jia.

**Methodology:** Rui Zhou, Ying Wang, Xukai Wang, Xukai Wang, Xiangyang Leng.

**Resources:** Rui Zhou.

**Software:** Rui Zhou.

**Supervision:** Yuyan Jia, Xukai Wang, Xiangyang Leng.

**Validation:** Rui Zhou, Yuyan Jia, Xukai Wang, Xiangyang Leng.

Writing – original draft: Rui Zhou.

Writing – review & editing: Rui Zhou.
